# Chimerism in the Realm of Hematopoietic Stem Cell Transplantation for Non-malignant Disorders—A Perspective

**DOI:** 10.3389/fimmu.2020.01791

**Published:** 2020-08-11

**Authors:** Clare Zimmerman, Shalini Shenoy

**Affiliations:** Department of Pediatrics, Washington University School of Medicine, St. Louis, MO, United States

**Keywords:** chimerism, chimerism after allo-HSCT, non-malignant diseases, hematopoeietic stem cell transplantation, bone marrow failure disorders, hemoglobinopathies, immunodeficiencies, metabolic disorder

## Abstract

Hematopoietic stem cell transplantation (HCT) is a curative intervention in non-malignant disorders (NMD) that benefit from donor-derived hematopoiesis, immunity, and establishment of vital cells or enzyme systems. Stability or reversal of disease symptoms depends on adequacy and long-term stability of donor cell engraftment in the compartment of interest. Unlike hematologic malignancies where complete replacement with donor derived hematopoiesis is desirable for a cure, NMD manifestations can often be controlled in the presence of mixed chimerism. This allows for exploration of reduced intensity conditioning regimens that can limit organ toxicity, late effects, and increase tolerability especially in young recipients or those with a large burden of disease related morbidity. However, the levels of donor chimerism conducive to disease control vary between NMD, need to focus on the hematopoietic lineage necessary to correct individual disorders, and need to be assessed for stability over time, i.e., a whole lifespan. An enhanced ability to reject grafts due to recipient immune competence, alloimmunization, and autoimmunity add to the complexity of this balance making NMD a highly diverse group of unrelated disorders. The addition of donor factors such as stem cell source and Human-Leukocyte-Antigen match extend the complexity such that ‘one size does not fit all'. In this perspective, we will discuss current knowledge of the role of chimerism and goals, approach to HCT, and emerging methods of boosting engraftment and graft function, and monitoring recommendations. We draw attention to knowledge gaps and areas of necessity for further research and research support.

## Introduction

Hematopoietic stem cell transplantation (HCT) is a curative option in a variety of inherited and acquired non-malignant disorders (NMD) that present at varying age groups, progress at variable rates, and have a wide range of clinical manifestations. These can vary from chronic supportive care needs and poor quality of life to rapid progression and early mortality. The overall aim of HCT is to correct the pathologic basis of hematologic, immunologic, or enzymatic dysfunction that is the etiology of the underlying disease. HCT results in immunohematologic replacement and thus corrects pathophysiology in NMD such as immune deficiencies and dysregulation, metabolic disorders, hemoglobinopathies, and bone marrow failure disorders. As identification of genetic abnormalities become more sophisticated, indications for curative transplant are expected to rise. As the number of disorders benefiting from transplant increase, expansion of donor sources that will best serve to mitigate disease manifestations is advantageous. Though HCT is curative, the risk-benefit ratio between disease manifestations and HCT outcomes should be carefully considered. The safety and efficacy of the procedure factors into the decision on how to, when to, and what to monitor post-transplant. Ensuring adequate and stable donor engraftment to effectively suppress disease manifestations or afford a cure in timely manner has a major role in determining outcomes and success when HCT is contemplated for NMD. Successful HCT does not guarantee reversal of non-hematopoietic abnormalities and outcomes are variable depending on disorder, graft source, conditioning regimen, toxicity, and other factors. We will focus on the chimerism aspect of success following HCT for NMD.

## Chimerism

Full donor chimerism is often unnecessary in NMD provided lineage specific engraftment is adequate to cure or suppress disease manifestations. However, a few scenarios give pause to this conclusion and disease specific assessment of chimerism requirement is necessary. Examples include metabolic neurodegenerative disorders where prompt and high percentage donor engraftment is required to stem neurodegeneration as early as possible and ensure an early rise in protective enzyme levels (1). Patients with inherited marrow failure disorders have a predisposition to myeloid malignancies. The stress of hematopoiesis has been implicated in malignant transformation, a stress relieved by successful HCT. It is not known whether this is adequate protection against transformation to leukemia in the setting of mixed chimerism post-HCT, making a case for regular long-term follow up in all HCT recipients.

### Frequency

Chimerism should be monitored at regular intervals after HCT. Though the duration of monitoring is variable, it is recommended that chimerism be monitored for at least 5 years post-HCT. The frequency of monitoring is higher in the first year post-HCT starting from the time of established engraftment, ~30 days post-HCT. In the presence of complete chimerism, monitoring every 3 months in the first year, 6 months in the second year and yearly for at least 5 years is a general guideline. In the event of unstable or mixed chimerism, intervals between testing should be shorter to determine additional interventions such as immunosuppression, stem cell boosts, or second transplant. Chimerism analysis should be paired with disease specific evaluations such as hemoglobin analysis in hemoglobinopathies, blood counts in marrow failure disorders, immune recovery in immunodeficiency disorders, and enzyme levels in metabolic disorders. In the event of poor marrow function it is important to determine whether the problem is due to lack of engraftment or poor graft function. Since etiology is often immune mediated in the former and a product of the marrow environment or donor source in the latter, the approach to investigation and mitigation vary. In general, a rapid drop of donor chimerism early post-HCT is difficult to halt. A gradual loss of engraftment over time may be more conducive to planning interventions that can help slow or prevent graft loss.

### Methods

Chimerism is detected by short tandem repeat (STR) polymerase chain reaction (PCR) analysis that quantifies donor and recipient DNA using individual specific repeats. It is read as a percentage of donor and recipient DNA in the sample, which can be peripheral blood or marrow. This procedure is the most sensitive and accurate method of testing. Pre-transplant samples from both donor and recipient are necessary for reporting post-HCT results. Alternate methods include florescent *in-situ* hybridization (FISH), chromosome analysis for sex chromosomes in the presence of sex discrepancy between donor and recipient, markers of donor hematopoiesis such as change in blood type, and a rise in previously absent enzyme levels in hereditary metabolic disorders. Disease specific testing such as neutrophil oxidative burst in chronic granulomatous disease or CD40 ligand expression in hyper-IgM syndrome can also assist in determining transplant efficacy. The latter tests are cheaper and can be used for screening but are not an accurate prediction of chimerism status.

### Lineage Specific Assessment

Most non-malignant disorders require lineage specific chimerism assessment which provides valuable detail despite the cost. Tracking chimerism in this manner allows for prediction of the role of chimerism on disease status and correction of deficit. Lineage specific evaluation is based on positive selection from peripheral blood cells and includes antibody column mediated cell separation into myeloid (CD15, CD33), T lymphoid (CD3), and B (CD19) lineage specificity followed by STR analysis. Additional less frequently used assessments of lineage specific chimerism include NK cells (CD16/CD56) or erythroid lineage cells (CD71) for appropriate disorders. Lower levels of mixed chimerism in non-essential lineages with stable full or adequate donor chimerism in the lineages of interest is capable of providing a cure. Serial tracking is necessary until stability is ensured.

In disorders where single lineage abnormalities cause disease, relevant lineage specific engraftment may be curative. For example, complete lymphoid engraftment with low myeloid chimerism (<50% donor) in Wiskott Aldrich syndrome can reverse the immune deficiency but not the thrombocytopenia. Serial determination of lineage specific chimerism can help predict impending rejection specific to each disease and facilitate earlier intervention (2).

Lineage specific chimerism requires an adequate number of lineage specific cells to determine chimerism levels. In the case of T-cell depleted transplants, reconstitution of the lymphoid compartment may be delayed. This may delay meaningful T-cell engraftment analyses. Similarly in the event of bone marrow suppression from factors such as infection, myeloid chimerism analyses may need to be performed after myeloid recovery.

## Disease Specific Chimerism

Children transplanted for NMD have a wide range of mixed chimerism where donor cell engraftment ranges from 33 to 78% (3–5). This variability is attributed to disease characteristics, immune competence, conditioning regimen, donor source, and transplant related complications. A retrospective review of an Italian cohort of 101 patients who underwent transplant for NMD found that chimerism remained a dynamic process where 55.4% of patients with early mixed chimerism post-HCT improved to only 12.8% with mixed chimerism at last follow-up (6). However, late graft failure though rare was still prevalent, making the case for continued follow-up. Early complete donor chimerism can correct disease manifestations sooner. However, early alloreactivity from donor lymphocyte engraftment can be associated with a higher incidence of both acute and chronic graft-versus-host (GVHD) disease. Mixed chimerism and gradual donor lymphoid engraftment invites tolerance and has a lower incidence of GVHD (6). A retrospective review of 56 patients transplanted for NMD showed that chimerism assessment on day +14 was significant in predicting 5-year event-free survival (EFS). It was higher in patients with complete or predominant donor chimerism compared to those with low-level mixed chimerism (86.1 vs. 71.4%, *p* < 0.001) (7). Our experience is similar in that a rapid decrease in donor engraftment early post-transplant suggests robust immunologic rejection and is harder to control without consideration of a second transplant.

The extensive variability in NMD makes it worthwhile to summarize chimerism studies by disease groups.

## Immune Deficiencies

Immune deficiencies that benefit from HCT are widely variable in the range of immune defects exhibited, some with additional hematologic manifestations. The most common disorders include severe combined immunodeficiency (SCID), Wiskott-Aldrich syndrome (WAS), and chronic granulomatous disease (CGD). The majority present during early childhood and SCID patients may be identified at birth with newborn screening. Immune dysregulation disorders that respond to treatment with HCT include hereditary hemophagocytic lymphohistiocytosis (HLH), immunodysregulation polyendocrinopathy enteropathy X-linked syndrome (IPEX), and autoimmune lymphoproliferative syndrome (ALPS). Non-SCID immune disorders are diagnosed at various ages most commonly due to infectious complications or hematopoietic/autoimmune manifestations. HCT provides the opportunity to establish a normal immune system but is a serious undertaking due to the risks associated with treating very young, or patients already exposed to serious infections. HCT is considered successful if patients demonstrate successful immune reconstitution in all lymphoid compartments, normal immunoglobulin levels, vaccine response, and T-cell repertoire. Achieving this in the presence of a dysregulated host immune system can be challenging given the propensity for selective engraftment and partial correction.

Patients with SCID have excellent survival rates post-HCT. Due to T-cell deficiency and the associated inability to reject grafts, for many years, the standard of care for SCID patients was to infuse donor stem cells without conditioning. This led to donor T-cell engraftment while other lineages remained of recipient origin resulting in lifelong dependency on immunoglobulin (IVIG) infusions due to a lack of functional B-cells in some SCID subtypes such as RAG1/2 deficiency.

Recently, retrospective reviews have shown that T-cell reconstitution was poorer with RAG and DCLRE1C mutations than other phenotypes. B-cell engraftment was poorer in IL2RG/JAK3, RAG and DCLRE1C mutation phenotypes especially with mismatched grafts (8, 9). With T-cell replete grafts, if at day +100, recipients had <300 CD3 cells/μL, <50 CD8 cells/μL, <10% CD45RA cells or a T cell repertoire of <13 of 24 families, a second HCT was likely needed (10). Global immune reconstitution has been successfully achieved in typical or leaky SCIDs transplanted after reduced intensity conditioning regimens. In contrast to T-cell engraftment, myeloid and B-cell reconstitution is improved by conditioning strength with myeloablative HCT affording better engraftment than reduced intensity HCT (11). Factors to be considered for toxicity of intensive regimens however include age of the patients, potential for late effects, pre-existing infections, and toxicity due to DNA repair defects (DCLRE1C). Another strategy to improve outcomes may be to avoid conditioning before an initial stem cell infusion if toxicity is imminent. This will need to be followed by a subsequent HCT with conditioning complete immune reconstitution when the recipient can tolerate it better.

All other immune deficiency disorders other than SCID require conditioning for engraftment. While myeloablative regimens have been associated with significant morbidity, reduced intensity regimens have better survival but higher rejection rates (12). Donor chimerism of >30% if stable, can protect against disease reactivation in HLH (13). Stable myeloid chimerism levels of >50% are desirable in conditions such as CGD for functional correction, and platelet count recovery in WAS. If familial donors are considered, the presence of heterozygosity for disease in the donor will need to be considered as it may interfere with full correction of the disease (e.g., HLH, X-linked CGD). Here, carrier donors are best avoided. Some immune dysregulation disorders such as STAT1 or CTLA-4 deficiency may require full donor chimerism for correction. Reducing the intensity or toxicity for safety may have secondary or late rejection rates of 10–15%, (14), autoimmunity with mixed chimerism (15) or delayed myeloid engraftment (16) and need to be tracked. Despite this, success rates continue to improve for these disorders.

## Metabolic and Storage Disorders

Metabolic and storage diseases are a heterogeneous group of disorders leading to accumulation of enzymatic by-products in multiple organs with associated toxicities, predominantly neurologic. HCT from unaffected donors can supplement enzyme following hematopoietic engraftment and migration to affected organs. Timely HCT is important to offset irreversible changes making early engraftment a key determinant of success.

Hurler Syndrome, a lysosomal storage disorder caused by deficiency of the enzyme alpha-L-iduronidase (IDUA) is the most commonly transplanted inherited metabolic disorder. Transplant is considered successful when patients attain normal leukocyte IDUA levels best achieved with full donor chimerism. In a study of more than 200 patients transplanted for Hurler's followed for neurodevelopmental outcomes and growth, factors supporting successful enzyme reconstitution included early transplantation, non-carrier donors, cord blood grafts due to higher inherent enzyme levels, and preparative regimens designed to achieve complete chimerism (1, 17). Speed of HCT for engraftment has no better illustration than early infantile Krabbe disease where donor engraftment following HCT <30 days of age had improved outcomes in domains of mobility, communication and feeding (18).

Leukodystrophies such as adrenoleukodystrophy (ALD), an X-linked disorder resulting in the inability to transport fatty acids into the peroxisome for degradation leads to accumulation in tissues specifically the central nervous system. HCT has been shown to halt neurological disease progression in a significant majority attributed to monocyte engraftment in the brain. It is important to proceed to transplant at diagnosis and with a low Loes radiologic score. Neurologic stability after engraftment takes time and occurs over the span of the first year so delays could accelerate deterioration. ALD patients with 70–100% donor chimerism in the myeloid compartment on day +60 and those with faster recovery of neutrophil counts had better resolution of gadolinium uptake on brain MRI scans post-HCT, a measure of blood brain barrier recovery (19).

Osteopetrosis, characterized by dysfunctional osteoclasts, also responds to HCT. Stabilization of vision and hearing, nasal obstruction and motor deterioration requires transplant in infancy and prompt engraftment is able to rescue 40–70% of patients. Graft failure despite intensive conditioning is the major cause of failure and death after HCT in osteopetrosis. However, donor chimerism in myeloid cells as low has 5–10% prevents death and provides sustained hematopoietic recovery in contrast to many other hereditary metabolic disorders (20).

## Hemoglobinopathies

Hereditary hemoglobinopathies with severe manifestations can be cured by HCT. The common disorders eligible for HCT are sickle cell disease (SCD) and transfusion dependent thalassemia. Variables influencing donor engraftment in hemoglobinopathy patients include age, HLA alloimmunization due to transfusion history, immune competence, and paucity of matched sibling donors. SCD manifestations are reversed in the presence of successful donor-derived erythropoiesis and normal erythroid precursors may have a survival advantage resulting in abatement of SCD symptoms even in the presence of low lymphoid engraftment. Myeloid lineage chimerism is a good surrogate for erythropoiesis in the absence of red cell chimerism (CD71) evaluation. Lymphoid engraftment can remain low or increase over time but there are no threshold levels necessary to maintain myeloid engraftment (21, 22).

Stable donor chimerism >20–25% paired with a hemoglobin S level <50% is associated with resolution of disease symptoms such as vaso-occlusive episodes and strokes (23, 24). However, hemolytic anemia was detected in SCD patients who had <50% donor cells after myeloablative conditioning and higher engraftment levels (>30%) were better if donors had sickle trait (25). The acceptability of stable mixed chimerism and the presence of mixed chimerism even with myeloablative conditioning (up to 44%) allows the exploration of less toxic regimens for HCT in SCD. Younger patients and donors, those with mismatched donors, low cell dose, and weaker stem cell sources such as cord blood have a higher risk of graft rejection in SCD and should be taken into consideration when fashioning regimens targeting intensity (26).

Thalassemia is cured when a patient no longer requires blood transfusions, regains growth, and restores iron related changes. A cohort of 106 patients with beta-thalassemia major when studied retrospectively revealed that half had sustained mixed donor chimerism with cure. Mixed chimerism was associated with a good transplant outcome and decreased risk of acute or chronic GVHD, a finding that was not noted in SCD. High erythroid lineage engraftment with low level donor chimerism in other lineages is compatible with cure in thalassemia (27). Mixed chimerism though acceptable, needs monitoring for stability over time in patients with hemoglobinopathy irrespective of intensity of conditioning regimens.

## Bone Marrow Failure Syndromes

Bone marrow failure syndromes (BMFS) such as aplastic anemia, severe congenital neutropenia, dyskeratosis congenita, Shwachman-Diamond, Diamond-Blackfan or Fanconi anemias (FA), and congenital amegakaryocytic thrombocytopenia include genetic and acquired pathologies resulting in inadequate hematopoiesis. HCT is curative. Since many are pre-leukemic conditions, full donor chimerism in the myeloid compartment is desirable and mixed chimerism in lymphoid lineages is acceptable. Patients with Fanconi and DNA repair defects are unable to tolerate regimen intensity or alkylating agents. The resulting need for balance between toxicity of intensification and engraftment is delicate. Graft rejection rates can be as high as 20–25% even with myeloablation. Both myeloablative and immunosuppressive regimens have been successful in curing BMFS following successful myeloid engraftment. In FA, with mixed chimerism in the lymphoid lineage, patients can be left with some lymphocytes exhibiting the increased sensitivity to DNA damage and others exhibiting a normal response (28–31). Long-term follow-up post-HCT in all BMFS should include both chimerism evaluation and monitoring for clonal hematopoiesis, a risk that should technically be mitigated with myeloid engraftment.

## Discussion

Donor chimerism requirements to achieve disease control are widely variable in NMD and can range from as low as 10% to >90% (Table 1). Variables influencing chimerism include age, inflammatory status, immune competence, and transfusion history. Transplant and donor characteristics include donor age, HLA match, stem cell source, cell dose, and intensity of conditioning regimens. In general, reduced intensity regimens are more appropriate for disorders that are conducive to mixed chimerism. As transplant approaches change to accommodate more donors, increase safety and tolerability, and reduce toxicity, chimerism should be tracked and described long-term.

**Table 1:** Classification of non-maligant disorders with associated lineage specific engraftment, and recommended donor chimerism levels for adequate disease mitigation.

**Non-malignant disorder**	**Lineage specificity**	**Minimum goal for donor Chimerism**
**Immunodeficiencies**		
HLH	NK cell/Lymphoid	>30% (13)
IPEX, ALPS	Lymphoid	>50% (32)
Severe Combined Immunodeficiency	T, B, NK cell	100% (8)
Chronic Granulomatous Disease	Myeloid	>50% (5)
Wiskott-Aldrich Syndrome	Lymphoid/Myeloid	>50% (16)
**Hemoglobinopathies**		
Sickle Cell Disease	Erythroid/myeloid	20–25% (23)
Thalassemias	Erythroid/myeloid	20–25% (27)
**Metabolic disorders**		
ALD, Hurlers, Krabbe's	Myeloid	70–100% (1)
Osteopetrosis	Myeloid	>10% (20)
**Bone marrow failure syndromes**		
SCN, SDS, DBA, FA	Myeloid	100% (30)
	Lymphoid	>50% (31)

Our understanding of chimerism and adequate interventions for the same continue to evolve. Stable donor chimerism off immunosuppression for over 2 years is unlikely to dwindle. However, continued monitoring is still recommended for occasional late graft rejections as described in thalassemia. These patients are usually identified by gradually dwindling donor chimerism levels in the lineage of interest. The old dogma that T-cell engraftment was necessary to maintain myeloid chimerism has not held true in NMD following the expanded ability to monitor chimerism in a lineage specific manner. While stable mixed chimerism is fully acceptable, dropping chimerism has prompted immune suppression withdrawal in myeloablated or immunoablated recipients whereas continuing immune suppression has been advantageous in low intensity regimens. Donor lymphocyte infusions are generally not of benefit, can induce unwanted GVHD, and should be avoided in NMD. An early rapid drop in chimerism usually requires a second stem cell infusion after reconditioning whereas a gradual decline can be salvageable with immune suppression adjustments. Reduced intensity conditioning does not preclude a second early transplant whereas a time lag is better for toxicity reasons after a myeloablative transplant. The ability to infuse products such as high dose CD34 selected stem cells is valuable in NMD to avert GVHD risks.

## Conclusions

The tracking of lineage specific donor chimerism for stability with time should be routinely incorporated into evaluations post-HCT for NMD. The definition of adequate chimerism for successful HCT varies by disorder and as our understanding of the same matures, our remedial interventions will evolve. The definition and durability of adequate chimerism has direct application to gene-modified therapy that is now under evaluation for many genetic disorders. In both the allogeneic and in the gene-modified autologous HCT setting, chimerism requirements will drive conditioning needs and transplant methods to achieve a cure.

## Data Availability Statement

The original contributions presented in the study are included in the article, further inquiries can be directed to the corresponding author.

## Author Contributions

All authors listed have made a substantial, direct and intellectual contribution to the work, and approved it for publication.

## Conflict of Interest

The authors declare that the research was conducted in the absence of any commercial or financial relationships that could be construed as a potential conflict of interest.
